# Awareness and Perceptions on Health Warning Labels on Cigarette Packs among Smokers: A Cross-Sectional Study

**DOI:** 10.1155/2020/9462903

**Published:** 2020-07-21

**Authors:** Thet Thet Hnin, Nang Naing Naing Shein, San Kyu Kyu Aye

**Affiliations:** ^1^Department of Preventive and Social Medicine, University of Medicine Mandalay, Mandalay, Myanmar; ^2^Mandalay Regional Department of Health, Mandalay Region, Department of Public Health, Ministry of Health and Sports, Mandalay, Myanmar; ^3^Department of Public Health, Ministry of Health and Sports, Naypyitaw, Myanmar

## Abstract

**Backgrounds:**

Tobacco use is the leading preventable cause of premature deaths. Tobacco control remains a top priority, and health warning labels (HWLs) are one of the recommended methods. This study is aimed at examining the awareness and perceptions of HWLs on cigarette packs among smokers.

**Methods:**

A cross-sectional study was conducted among 240 smokers who were randomly recruited from three townships in Mandalay in 2018. A face-to-face interview was done using a questionnaire. Multivariate logistic regression was used to analyse the data.

**Results:**

About half were 18-40 years old; the majority were males (96.3%) and smokers (93.4%). Nearly all respondents noticed both pictorial warning and text messages, and about half could identify the current size of HWLs. Most of the smokers generally had positive perceptions and opinions on HWLS, and they strongly supported it. About 75% intended to reduce the number of cigarettes, and 18% were willing to quit within 6 months. Those who desired to reduce the number of cigarettes were more likely to quit within 6 months (aOR = 7.6, 95% CI 1.6-35.9 and aOR = 19.6, 95% CI 13.0-294.7 for those who had a little and strong desire, respectively).

**Conclusion:**

Awareness status and perceptions of the respondents were acceptable, and HWLs have motivated smokers to quit smoking. The Tobacco Control Program needs to strengthen the tobacco control law that prohibits selling loosies in order to maximize the benefits of HWLs.

## 1. Introduction

Globally, about 8 million deaths are related to tobacco each year, and more than 80% of the world's tobacco users (1.3 billion) are from developing countries [1]. Tobacco-related deaths were more than those who died of other infectious diseases such as tuberculosis, malaria, and acquired immune-deficiency syndrome [2]. Tobacco use actually is the single greatest preventable cause of premature deaths [3]. It is also one of the main risk factors for noncommunicable diseases, including lung cancer, oral cancer, esophageal cancer, cardiovascular diseases, and chronic obstructive pulmonary diseases [4]. Among Southeast Asian countries, Myanmar is one of the countries with high prevalence of tobacco use with prevalence among males aged 15 and above is 43.8% and among females is 8.4% [5]. Moreover, the smoking prevalence among youths (13-15 years) of Myanmar is 10.6% [6]. Smokers purchase some proportion of their income on tobacco, and smoking-related illnesses increase out-of-pocket payment for health care. This can lead to significant financial burden on families, especially the poor [7, 8]. Moreover, tobacco use becomes a major public health problem and brings a serious negative impact on the country due to increased health care expenditure, premature deaths, and lost productivity [3, 7, 8].

Tobacco control is a multisectorial approach, and it remains top priority of the public health program [2]. Health warning labels (HWLs) on cigarette packages have appeared to be one of the recommended public health measures for disseminating harms of tobacco products to public, especially to smokers in order to persuade them to reduce or stop consumption. Moreover, HWLs also discourage from experimentation and initiation of tobacco products [9–12]. HWLs on cigarette packages could offer high frequency of exposure to health messages, and pack-a-day smokers are possibly exposed to HWLs about 7,000 times in a year [13]. Effective warning labels should be clear, visible, readable, and rotating and probably covered at least 50% of the front and back of packages with shocking images [11, 12]. In addition, HWLs with larger prominent images are superior compared to smaller ones [4], and text-only messages [4, 14, 15] and frequently changing images could sustain effectiveness of HWLs over time [14].

HWL is generally a cost-effective intervention to decrease smoking [16]. The previous studies in high-income countries found that pictorial warnings increased health knowledge on smoking, perceptions on risks of smoking, and likelihood of cessation among smokers [17–19]. A similar finding was found in low- and middle- -income countries as well [20–24]. However, other studies showed that the warning labels have no effect on intention to quit [25, 26]. The impact of HWLs varies across different cultures and geographical areas [4], and perceptions on risks of smoking are usually influenced by sociodemographic characteristics of people [27, 28].

Myanmar approved legislation for HWLs on cigarette packs, and the law came into effect on 1 September 2016. It is required to cover 75% of the front and back surfaces of tobacco product packing, in which the image is to cover 50% and the text message is to cover 25%. Ten images with corresponding text messages are ordered for printing on packages. Each HWL is used for one year, and it will be rotated to the next one in the series. This will be also applied to local products (cheroot packages) [29]. To the best of our knowledge, in Myanmar, there were many studies related to tobacco [30–32], but very few studies about HWLs were conducted. This study therefore is aimed at examining the smokers' awareness and perceptions on HWLs on cigarette packs.

## 2. Materials and Methods

### 2.1. Study Design and Sampling Procedure

A community-based cross-sectional study was conducted in Mandalay City from May to August 2018. This study was approved by the Ethical Review Committee of the University of Medicine, Mandalay, Myanmar, with ID No.170 MPTM/UMM/2018.

Smokers aged 18 and older who smoked at least 100 cigarettes in their lifetimes and lived in Mandalay City during data collection were included. There are seven townships in Mandalay City, and each township has about 18 wards or quarters. Firstly, three townships were selected from the seven townships by a lottery method. Secondly, two wards were selected from the selected townships by simple random sampling. Then, we totally got six wards. The list of the household having at least one smoker from the selected wards was collected by a researcher and other data collectors together with the local authorities and basic health staff. Finally, 40 smokers were selected from each ward by systematic random sampling. The total number of sample size was 240.

If there was more than one eligible person in the selected household, the smoker was selected by a lottery method. If the selected smoker was not available during the first time of data collection, he or she was interviewed another time.

### 2.2. Data Collection Procedures

After getting informed consent, five to ten smokers were recruited each day by face-to-face interview using a structured questionnaire. The questionnaire was adopted from the previous study [21]. The questionnaire was pretested and modified as required in order to improve clarification.

### 2.3. Variables

The questionnaire included three parts: sociodemographic and personal characteristics of smokers, awareness of HWLs, and perceptions and opinions on HWLs on cigarette packs. Questions about the smoker's sociodemography were age, gender, level of education, and monthly income. Questions related to their personal characteristics were smoking status, type of tobacco products, duration of smoking, and amount of tobacco consumption per day. Smoking status was categorized as a current smoker who currently smokes cigarettes or who quits smoking within 3 months at the time of data collection and an ex-smoker who stops smoking more than 3 months at the time of the interview. Awareness of HWLs was assessed by sites, sizes, and types of HWLs and contents of text messages. Perceptions on HWLs included health risks of smoking, arousing fear of smoking, determination to quit, and opinions on the effectiveness of HWLs on young people and illiterate smokers and implementation of HWLs. The responses were self-reported rating of smokers' perception: strongly support, somewhat support, somewhat oppose, and strongly oppose and very effective, effective, ineffective, and very ineffective.

Our main outcome variable was intention to quit. Smokers were asked, “Do you have a plan to quit smoking when you see HWLs on cigarette pack?,” and they could respond as “Yes” or “No.” If they replied “Yes,” they would be asked the estimated duration to quit. The possible answers were (1) within 1 month, (2) 1-6 months, and (3) >6 months.

### 2.4. Data Analysis

After data collection, the completeness and consistency of data were checked daily. Data analysis was done by using Stata 13 (Stata Corp, College Station, TX). Complex sampling design and sampling weights were adjusted during data analysis. Data summarization for categorical data was done as frequency tables, and continuous data was described as the mean and standard deviation. The association between the smoker's sociodemographic characteristics, smoking status, awareness and perceptions on HWLs, and intention to quit was determined by using univariate and multivariate logistic regression. Independent variables with a *P* value of <0.15 in univariate analysis were included in the multivariate model. *P* value < 0.05 was considered to be statistically significant.

## 3. Results

This study included a total of 240 respondents aged 18 and above who smoked at least 100 cigarettes in their lifetimes. The youngest was 18 years and the oldest was 85 years, and 30.3% were younger than 29 years. The vast majority were males (96.3%) and mostly attained middle school (33.3%) and high school (30.1%). About 5.6% earned less than MMK 100,000 (USD 66) per month, and only 31.7% got monthly income more than MMK 300,000 (USD 200). The majority were current smokers (93.4%) and mostly smoked more than 10 years (60.0%) and smoked 1-5 cigarettes per day (59.0%). Most of them usually smoked cigarettes (82.0%) and cheroots (71.7%) which are traditional hand-rolled thin cigars (Table 1).

Almost all of the respondents (98.4%) were aware of HWLs on the front of the cigarette packs but 68.4% wrongly answered on the top of the packs. Only 16.6% were always aware of HWLs while mostly (72.8%) were sometimes aware of it. Less than half of the respondents (46.7%) could correctly identify the size of the HWLs which is three-fourths of the pack. Almost all (99.4%) noticed the text messages on the packs and mostly stated that smoking can cause heart and lung diseases (67.7%), followed by the statements that smoking can cause cancers (41.8%) and smoking can worsen your health (37.5%) (Table 2).

Fifty-two percent of smokers believed that HWLs can improve health knowledge just a little and 19.8% did not believe so. After seeing HWLs, 38.3% thought a little about dangers of smoking and 36.6% felt a little fear of smoking, while 22.4% did not think so and 33.1% did not feel so. About 75.0% considered to reduce the number of cigarettes smoked per day, and 18.3% desired to quit within 6 months. The majority of smokers suggested that HWLs are effective for young people (75.6%) and illiterate smokers (89.3%). About half of the respondents strongly (47.6%) and somewhat (44.4%) supported HWLs (Table 3).

It showed that desire to reduce the number of cigarettes smoked per day (a little or a lot) was only significantly associated with intention to quit (aOR = 7.6, 96% CI 1.6-35.9 and aOR = 19.6, 95% CI 13.0-294.7, respectively) after adjusting other covariates. Smokers with positive perceptions on HWLs and those with strong support of HWLs were more likely to intend to quit smoking than their counterparts, but the association was not statistically significant. None of the sociodemographic and personal characteristics were associated with desire to quit (Table 3).

## 4. Discussion

Among smokers, almost all of the respondents were aware of HWLs on cigarette packs, and they generally have positive perceptions and opinions on HWLs. Smokers who have desire to reduce the number of cigarettes smoked per day have a significant intention to quit smoking within 6 months.

In our study, the vast majority of smokers were aware of HWLs. The early studies in India showed that 82% of smokers [20] and 72% of tobacco consumers had ever noticed the warning on cigarette packs [33]. It means that smokers are more aware of HWLs on cigarette packs, and it might help them to remind the health risks of smoking whenever they smoke.

Awareness of HWLs on the front, side, and back of the packs was considerably high among smokers. On the other hand, some smokers wrongly mentioned that it is on the top of the packs which is not. The participants in Laos described that they were more aware of health information on the side of the cigarette packs (66%) than on the back (12%) and front of the packs (19%) [21]. The different results are probably related to differences in characteristics of the study population; for instance, only smokers were included in the present study while both smokers and nonsmokers were involved in the latter study. Moreover, HWLs were different between the two countries. In most countries, cigarette packages have featured HWLs, but these vary in the text and graphic messages (see the latest number of countries implementing HWLs on cigarette packs https://tobaccolabels.ca/healthwarningsinfo/).

Almost all of our study participants noticed text message warning on cigarette packs which is slightly higher than that in Malaysia (81% noticed the warning messages) [25]. Different types of messages were found in the studies. In our study, smokers mentioned that smoking can cause heart and lung diseases, smoking can cause cancers, and smoking can worsen one's health. In the study in Malaysia, the participants answered that smoking causes lung cancer (43%), prematurity (41%), gangrene (38%), miscarriage (33%), and so on [25]. This is reasonable and might be simply due to the fact that different countries use different types of questions and different types of warning messages on cigarette packs at the time of data collection. It also revealed that the contents of the health messages largely depend on the local context.

Evidences suggest that the size and design of pictorial warnings are related to the desired effects such as encouraging cessation and discouraging smoking [4]. In addition, the World Health Organization Framework Convention Tobacco Control recommends that HWLs should cover 50% of product packages [12]. Nearly half of the respondents in this study were able to observe the correct size of HWLs currently used in our country. This indicates they looked closely at it. The study in Canada stated that 79% read the warning labels at least once [13]. Similarly, more than 75% of Malaysian smokers had noticed and read HWLs closely [23]. The consistency of findings indicates that HWLs are useful to raise smokers' awareness. Evidences also suggest that HWLs on cigarette packages increase the number of exposure of health messages to smokers and are a more direct way to communicate with them [4, 13]. However, the wide availability of loosies may reduce the effectiveness of HWLs.

Our findings described that some smokers had a positive perception and strong opinion on HWLs in terms of improving health knowledge, arousing fear of smoking, and thinking about harms and risks of smoking. Similarly, the participants from the early studies also found that HWLs can improve people knowledge a lot [21], they can evoke fears of smoking [22], and they can remind harms and risks of smoking [15, 21, 34]. These findings highlight that although some participants had a positive perception of HWLs, there is a wide variation of levels of individuals' perceptions and opinions among people. This might be due to different personal characteristics, individuals' preexisting beliefs, and perceptions.

It is widely recognised that smokers desire to reduce the number of cigarettes and to quit due to HWLs. However, the number of smokers with such intention varies in different studies. In the present study, most of the current smokers desired to reduce the stick of cigarettes and about one in five were willing to quit within 6 months. A similar finding in India reported that about 15% thought to quit smoking [33]. Likewise, one-third of smokers in Jordan stopped smoking because of HWLs [22]. More than 40% of Canadian smokers [18] and 56% of Malaysian smokers [23] also reported that the pictorial warnings have motivated them to quit. A much higher number of smokers (80%) stated that they will reduce the number of cigarettes and more than half of them said that they will quit within six months among Indian smokers [20]. Although smokers were willing to quit, they still had some delay in quitting. With regard to this, social norms and individuals' perceptions and attitudes might probably influence their desire a lot [4].

HWL is an easy and economical method to create awareness among illiterate people [20]. Among Canadian youths,90% agreed that HWLs on cigarette packages are able to disseminate health risks of smoking and stimulate them to think about smoking being less attractive [18]. In Korea, about 70% of adolescents reported the same and the association was stronger if they have received other antitobacco activities [35]. Among French youths, HWLs significantly aroused fear and harm of smoking and it was associated with decrease initiation of smoking [36]. Additionally, nonsmokers also mentioned that HWLs have motivated them to discourage to smoke [22]. The majority of the respondents perceived that HWLs are an effective way to disseminate health information to low literacy smokers and young people. Although our findings could not directly reflect the target group's opinions because all participants were adults, it is highly acceptable. Therefore, HWLs will probably be a useful tool for transferring health messages to illiterate people, nonsmokers, and youths.

HWLs have a reasonable level of support even by smokers themselves. We found that some of the respondents strongly supported HWLs. Previous studies also showed that both smokers and nonsmokers suggested that HWLs on tobacco packs were very important and useful [15, 22].

Smokers who desired a little or a lot to reduce the number of cigarettes were significantly associated with intention to quit smoking. Those who had positive perceptions of HWLs in different ways and those who supported HWLs were more likely to consider quitting than those who did not. A review in Asia described that HWLs decreased initiation of smoking among nonsmokers and increased intention to quit among smokers effectively [37]. A trial in the US showed that pictorial warning was associated with intention to quit smoking over 4 weeks [34]. Smokers who thought harms (aOR = 1.7), quit-likely (aOR = 1.8), and foregoing cigarettes (aOR = 2.0) due to HWLs were significantly associated with intention to quit [23]. These results indicate that individuals' perceptions and experiences, social norms, and geographical areas greatly influence the impact of HWLs [4].

### 4.1. Strengths and Limitations

This study has some factors that can be taken as its strength. The findings of this study can be generalized for smokers who lived in urban cities because the respondents were randomly selected. This is the first study to explore awareness and perceptions of HWLs in Myanmar after the implementation of HWLs on cigarette packs. Our study informs the Tobacco Control Program in Myanmar about the effectiveness of HWLs. However, our study also has some limitations. Generalizability of the findings could be limited for smokers in rural areas, nonsmokers, and youths because this study was conducted in an urban area and involved only adult smokers who smoked at least 100 cigarettes in their lifetime. By design, recall bias could not be excluded from this study. However, this is a feasible method of data collection in the community.

## 5. Conclusion

This study suggests that the current HWL is effective for disseminating health risks of smoking and it can trigger positive perceptions and opinions on HWLs among smokers. However, retail sales outlets will probably limit its effectiveness because smokers cannot see the warning messages when buying loosies. Therefore, the Tobacco Control Program in Myanmar needs to strengthen the tobacco control law enforcement that prohibits selling loosies in order to achieve the intended benefits of HWLs. The program also needs to provide cessation support services for those who are willing to quit. Further research including nonsmokers and rural population are recommended to get generalizability for the whole community. Moreover, a longitudinal study design is also suggested to assess how many smokers will quit among those who expressed intention to quit.

## Figures and Tables

**Table 1:** Sociodemographic and personal characteristics of smokers.

Characteristics	Frequency	Percent	95% CI
Age group (year) (*n* = 240)			
≤29	69	30.3	19.4-44.1
30-39	48	18.6	7.7-38.4
40-49	49	23.3	8.9-48.6
50-59	38	14.5	6.7–28.6
60-69	23	9.0	5.2–15.2
≥70	13	4.3	0.5–28.5
Gender (*n* = 240)			
Male	229	96.3	70.1–99.7
Female	11	3.7	0.3–29.9
Level of education (*n* = 240)			
Illiterate	4	1.2	0.02-8.0
Read and write	14	5.9	3.1-10.9
Primary school	53	20.1	8.0–42.1
Middle school	79	33.3	23.2–45.4
High school	69	30.1	19.7–42.5
Diploma to postgraduate	21	9.4	4.0–20.4
Income (MMK)^∗^ (*n* = 208)			
<100000	13	5.6	1.8–15.7
100000-199999	74	36.7	21.5–55.1
200000-299999	55	26.0	8.1–58.2
≥300000	66	31.7	18.7–48.4
Smoking status (*n* = 240)			
Current smoker	225	93.4	80.5–98.0
Ex-smoker	15	6.6	1.9–19.5
Duration of smoking (years) (*n* = 225)			
1-5	57	26.0	17.4–37.0
6-10	32	14.0	10.6–18.2
>10	136	60.0	51.3–68.0
Number of tobacco smoked per day (*n* = 225)			
1-5 sticks	123	59.0	40.8–75.1
>5 sticks	102	41.0	24.9–59.2
Types of tobacco^∗∗^ (*n* = 225)			
Cigarette	187	82.0	67.7–90.7
Cheroot	159	71.7	62.0–79.8
Hand-rolled cheroot	12	2.7	1.1–6.5
Cigar	6	4.6	0.6–26.5

MMK = kyats; ^∗^1 USD = MMK 1500 (exchange rate at the time of interview). ^∗∗^Multiple responses.

**Table 2:** Awareness of health warning labels on cigarette packs among smokers.

Characteristics	Frequency	Percent
Sites of HWLs^∗^ (*n* = 240)		
Front	236	98.4
Side	214	85.3
Back	205	88.8
Top	170	68.4
Frequency of notice of HWLs (*n* = 240)		
Always	39	16.6
Usually	24	10.6
Sometimes	177	72.8
Percentage of HWLs (*n* = 240)		
One-fourth of pack (25%)	7	3.5
Half of pack (50%)	58	23.7
Three-fourth of pack (75%)	109	46.2
Full pack (100%)	66	26.6
Awareness of warning messages (*n* = 240)		
Yes	238	99.4
No	2	0.6
Types of health messages^∗^ (*n* = 238)		
Smoking can worsen your health	161	37.5
Do not sell cigarette under 18 years	95	12.7
Smoking can cause oral cancer	85	3.5
Smoking can cause heart and lung diseases	26	67.7
Smoking can cause cancers	6	41.8

^∗^Multiple responses.

**Table 3:** Crude and adjusted odds ratios for intention to quit among smokers with their perceptions of health warning labels on cigarette packs (*n* = 225).

Perception and opinion	Frequency (%)	OR (95% CI)	aOR (95% CI)
Improve health knowledge			
Not at all	41 (19.8)	Reference	
A little	114 (51.3)	3.9 (0.3-47.1)	1.3 (0.2-9.6)
Somewhat	52 (21.9)	8.1 (1.2-56.9)	1.4 (0.2-13.8)
A lot	18 (7.0)	11.2 (0.5-239.8)	2.7 (0.03-202.6)
Thinking about health risks of smoking			
Not at all	48 (22.4)	Reference	
A little	84 (38.3)	3.5 (1.7-7.5)	1.4 (0.5-4.4)
Somewhat	64 (28.0)	9.6 (0.5-199.0)	2.4 (0.5-4.4)
A lot	29 (11.3)	5.7 (0.2-139.0)	0.5 (0.01-21.3)
Arousal of fear of smoking			
Not at all	70 (33.1)	Reference	
A little	83 (36.6)	3.8 (0.9-16.2)	1.2 (0.2-7.7)
Somewhat	50 (20.5)	5.8 (1.1-30.0)	1.7 (0.2-17.4)
A lot	22 (9.7)	14.6 (0.2-114.8)	4.2 (0.2-96.0)
Desire to reduce the number of cigarettes per day			
Not at all	55 (25.7)	Reference	
A little	108 (49.1)	9.3 (3.2-27.0)	7.6 (1.6-35.9)
Somewhat	41 (16.9)	9.6 (1.0-87.6)	4.5 (0.7-26.7)
A lot	21 (8.3)	50.5 (1.0-248.5)	19.6 (13.0-294.7)
Effectiveness of HWLs on young people			
Ineffective	55 (24.4)	Reference	
Effective	125 (55.6)	2.2 (0.5-9.3)	1.0 (0.2-4.0)
Very effective	45 (20.0)	3.3 (0.4-29.5)	1.3 (0.1-26.1)
Effectiveness of HWLs on illiterate people			
Ineffective	24 (10.7)	Reference	
Effective	135 (60.0)	3.3 (1.1-10.4)	2.2 (0.2-21.0)
Very effective	66 (29.3)	5.6 (1.1-28.5)	2.3 (0.1-41.1)
Support of HWLs			
Somewhat oppose	18 (8.0)	Reference	
Somewhat support	100 (44.4)	2.0 (0.3-12.2)	1.6 (0.06-45.5)
Strongly support	107 (47.6)	3.9 (0.4-39.1)	1.7 (0.03-106.3)

## Data Availability

The data used to support the study findings are mostly included in the article. It is available from the corresponding author upon request.

## Supplementary Materials

Supplementary Materials Questionnaire of awareness and perceptions on health warning labels on cigarette packs among smokers.

## References

[1] WHO (2020). *Tobacco*.

[2] Narain J. P., Sinha D. N. (2011). Tobacco epidemic in South-East Asia region: challenges and progress in its control. *Indian Journal of Public Health*.

[3] WHO (2016). *The Tobacco Atlas*.

[4] Hammond D. (2011). Health warning messages on tobacco products: a review. *Tobacco Control*.

[5] WHO (2019). *WHO report on the Global Tobacco Epidemic 2019*.

[6] WHO (2016). *Myanmar Global Youth Tobacco Survey 2016*.

[7] Kyaing N. N., Sinha D. N., Islam M. A., Rinchen S. (2011). Social, economic and legal dimensions of tobacco and its control in South-East Asia region. *Indian Journal of Public Health*.

[8] Ekpu V. U., Brown A. K. (2015). The economic impact of smoking and of reducing smoking prevalence: review of evidence. *Tobacco Use Insights*.

[9] Slade J. (1997). The pack as advertisement. *Tobacco Control*.

[10] Wakefield M., Morley C., Horan J. K., Cummings K. M. (2002). The cigarette pack as image: new evidence from tobacco industry documents. *Tobacco Control*.

[11] World Health Organization (2008). *Guidelines for implementation of Article 11 of the WHO Framework Convention on Tobacco Control (packaging and labelling of tobacco products)*.

[12] WHO (2003). *WHO Framework Convention on Tobacco Control*.

[13] Hammond D., Fong G. T., McDonald P. W., Cameron R., Brown K. S. (2003). Impact of the graphic Canadian warning labels on adult smoking behaviour. *Tobacco Control*.

[14] Woelbert E., d’Hombres B. (2019). Pictorial health warnings and wear-out effects: evidence from a web experiment in 10 European countries. *Tobacco Control*.

[15] Chung-Hall J., Fong G. T., Meng G. (2020). Effectiveness of text-only cigarette health warnings in Japan: findings from the 2018 International Tobacco Control (ITC) Japan Survey. *International Journal of Environmental Research and Public Health*.

[16] Hiilamo H., Crosbie E., Glantz S. A. (2014). The evolution of health warning labels on cigarette packs: the role of precedents, and tobacco industry strategies to block diffusion. *Tobacco Control*.

[17] Borland R., Wilson N., Fong G. T. (2009). Impact of graphic and text warnings on cigarette packs: findings from four countries over five years. *Tobacco Control*.

[18] Hammond D., Fong G. T., Borland R., Cummings K. M., McNeill A., Driezen P. (2007). Text and graphic warnings on cigarette packages: findings from the international tobacco control four country study. *American Journal of Preventive Medicine*.

[19] O’Hegarty M., Pederson L. L., Nelson D. E., Mowery P., Gable J. M., Wortley P. (2006). Reactions of young adult smokers to warning labels on cigarette packages. *American Journal of Preventive Medicine*.

[20] Mangat D. S., Mangat H. S., Singh K. (2017). Impact of pictorial health warning labels on people consuming tobacco products in smoking form. *Journal of Advanced Medical and Dental Sciences Research*.

[21] Sychareun V., Hansana V., Phengsavanh A., Chaleunvong K., Tomson T. (2015). Perceptions and acceptability of pictorial health warning labels vs text only - a cross-sectional study in Lao PDR. *BMC Public Health*.

[22] Bader R. K., Shihab R. A., al-Rimawi D. H., Hawari F. I. (2018). Informing tobacco control policy in Jordan: assessing the effectiveness of pictorial warning labels on cigarette packs. *BMC Public Health*.

[23] Fathelrahman A. I., Omar M., Awang R., Cummings K. M., Borland R., Samin A. S. B. M. (2010). Impact of the new Malaysian cigarette pack warnings on smokers’ awareness of health risks and interest in quitting smoking. *International Journal of Environmental Research and Public Health*.

[24] Reid J., Mutti-Packer S., Gupta P. (2017). Influence of health warnings on beliefs about the health effects of cigarette smoking, in the context of an experimental study in four Asian countries. *International Journal of Environmental Research and Public Health*.

[25] Rahman M. M., Arif M. T., Abd R. M. (2015). Effectiveness of pictorial health warning on cigarette packages: a cross-sectional study in Sarawak, Malaysia. *Malaysian Family Physician: the official journal of the Academy of Family Physicians of Malaysia*.

[26] Drovandi A. D., Teague P.-A., Glass B. D., Malau-Aduli B. S. (2018). Australian school student perceptions of effective anti-tobacco health warnings. *Frontiers in Public Health*.

[27] Siahpush M., McNeill A., Hammond D., Fong G. (2006). Socioeconomic and country variations in knowledge of health risks of tobacco smoking and toxic constituents of smoke: results from the 2002 International Tobacco Control (ITC) Four Country Survey. *Tobacco Control*.

[28] Wang Q., Shen J. J., Sotero M., Li C. A., Hou Z. (2018). Income, occupation and education: are they related to smoking behaviors in China?. *PloS One*.

[29] Tun A. (2019). *World No Tobacco Day 2019: improving health, boosting economy by engaging in smoking control in Myanmar*.

[30] Tun N. A., Chittin T., Agarwal N., New M. L., Thaung Y., Phyo P. P. (2017). Tobacco use among young adolescents in Myanmar: findings from global youth tobacco survey. *Indian Journal of Public Health*.

[31] Latt N. N., Saw Y. M., Cho S. M., Kariya T., Yamamoto E., Hamajima N. (2018). Tobacco Control Law awareness, enforcement, and compliance among high school students in Myanmar. *Nagoya Journal of Medical Science*.

[32] Myint M. N. H. A., Yamamoto E., Ko M. H., Khaing M., Reyer J. A., Hamajima N. (2019). Knowledge, attitude, and usage pattern of tobacco among high school students in Nay Pyi Taw, Myanmar. *Nagoya Journal of Medical Science*.

[33] Karinagannanavar A., Raghavendra B., Hemagiri K., Goud T. G. (2011). Awareness about pictorial warnings on tobacco products and its impact on tobacco consumers in Bellary, India. *Asian Pacific Journal of Cancer Prevention*.

[34] Brewer N. T., Hall M. G., Noar S. M. (2016). Effect of pictorial cigarette pack warnings on changes in smoking behavior: a randomized clinical trial. *JAMA Internal Medicine*.

[35] Hwang J.-e., Cho S.-i. (2020). The association between new graphic health warning labels on tobacco products and attitudes toward smoking among south Korean adolescents: a national cross-sectional study. *BMC Public Health*.

[36] Lesueur F. E.-K., Bolze C., Gomajee R., White V., Melchior M. (2019). Plain tobacco packaging, increased graphic health warnings and adolescents’ perceptions and initiation of smoking: DePICT, a French nationwide study. *Tobacco Control*.

[37] Ratih S. P., Susanna D. (2018). Perceived effectiveness of pictorial health warnings on changes in smoking behaviour in Asia: a literature review. *BMC Public Health*.

