# Supplementary material for: Awareness and Perceptions on Health Warning Labels on Cigarette Packs among Smokers: A Cross-Sectional Study
Source: Biomed Res Int. 2020 Jul 21;2020:9462903. doi: 10.1155/2020/9462903 (PMC7391087; doi:10.1155/2020/9462903)
Supplement: Supplementary Materials — Questionnaire of awareness and perceptions on health warning labels on cigarette packs among smokers. [file 9462903.f1.pdf]

# Questionnaire

## Questionnaire for acquiring information about awareness and perceptions on health warning labels on cigarette packs among smokers

Date ☐☐☐☐ Serial No. ☐☐☐☐

### Section A. Socio-demographic and personal characteristics of smokers

| No. | Questions | Code |
|---|---|---|
| D1 | Age in completed years ------------ | ☐☐ |
| D2 | Gender<br>(1) Male (2) Female | ☐ |
| D3 | Education<br>(1) Illiterate<br>(2) Read and write<br>(3) Primary school certificate<br>(4) Middle school certificate<br>(5) High school certificate<br>(6) Graduate/Diploma<br>(7) Others (specify) ………… | ☐ |
| D4 | How much monthly income do you get? (Kyats) ----------------------- | ☐ |

### Section B. Smoking status

| No. | Questions | Code |
|---|---|---|
| S1 | Are you currently smoked?<br>(1) Yes **(Skip S2)** (2) No | ☐ |
| S2 | If not, how long have you been stop? **(Go to Section C)**<br>(1) < 3months<br>(2) > 3months | ☐ |
| S3 | **For Current smoker**<br>If yes, which of these products are you using now?<br>(1) Cigarette 1. Yes 2. No<br>(2) Cheroot 1. Yes 2. No<br>(3) Cigar 1. Yes 2. No<br>(4) Hand-rolled cheroot 1. Yes 2. No<br>(5) Others (specify) ………………………. | ☐<br>☐<br>☐<br>☐<br>☐ |
| S4 | How long have you been smoking? ---------------- | ☐ |
| S5 | How many sticks did you smoke per day? -------------- | ☐ |

## Section C. Awareness of pictorial health warnings on cigarette packs

| No. | Questions | Code |
|---|---|---|
| A1 | Which side contains pictorial health warnings on cigarette packs?<br>(1) Front<br>(2) Back<br>(3) Side<br>(4) Top<br>(5) Don't know | ☐<br>☐<br>☐<br>☐ |
| A2 | How often do you notice the health warnings on the cigarette packs?<br>(1) Always<br>(2) Usually<br>(3) Sometimes | ☐ |
| A3 | How much percentage of health warning occupy on the cigarette packs?<br>(1) 25% (One-fourth of pack)<br>(2) 50% (Half of pack)<br>(3) 70% (Three-fourth of pack)<br>(4) 100% (Full pack)<br>(5) Others (specify) …………. | ☐ |
| A4 | Do you aware the health warnings text on cigarette packs?<br>(1) Yes (2) No | ☐ |
| A5 | What type of health message on cigarette packs?<br>(1) Smoking can cause oral cancer<br>(2) Do not sell cigarette under 18 years<br>(3) Smoking can cause heart disease and lung diseases<br>(4) Nitrosamines, benzopyrene etc., including in cigarettes can cause cancers<br>(5) Smoking can worse your health<br>(6) Others (specify)…………. | ☐<br>☐<br>☐<br>☐<br>☐<br>☐ |

## Section D. Perceptions and opinions on health warning labels on cigarette packs

| No. | Questions | Code |
|---|---|---|
| P1 | Would you say the inclusion of health warnings and health message on cigarette packs has improved your knowledge of the health effects of smoking?<br>(1) Not at all<br>(2) A little<br>(3) Somewhat<br>(4) A lot | ☐ |
| P2 | To what extent, do the health warnings make you think about the health risks or dangers of smoking?<br>(1) Not at all<br>(2) A little<br>(3) Somewhat<br>(4) A lot | ☐ |
| P3 | To what extent, do the health warnings make you in arousing fear of smoking?<br>(1) Not at all<br>(2) A little<br>(3) Somewhat<br>(4) A lot | ☐ |
| P4 | In terms of the way you feel about your own smoking behavior, how much do you think the health warnings on cigarette packs can help you smoke less per day?<br>(1) Not at all<br>(2) A little<br>(3) Somewhat<br>(4) A lot | ☐ |

| No. | Questions | Code |
|---|---|---|
| P5 | Do you have a plan to quit smoking when you see HWLs on cigarette packs?<br>(1) Yes (2) No | |
| P6 | If YES and you consider completely quitting smoking, are you going to quit seriously in the next?<br>(1) Within 1 month<br>(2) 1-6 months<br>(3) > 6 months<br>(4) Others (specify)………… | |
| P7 | To what extent, the effectiveness of health warning on cigarette packs can prevent smoking among young people?<br>(1) Very effective<br>(2) Effective<br>(3) Ineffective<br>(4) Very ineffective | |
| P8 | To what extent, health warnings on cigarette packs will have greater impact on low literacy smokers?<br>(1) Very effective<br>(2) Effective<br>(3) Ineffective<br>(4) Very ineffective | |
| P9 | How much do you support the implementation of health warnings on cigarette packs?<br>(1) Strongly support<br>(2) Somewhat support<br>(3) Strongly oppose<br>(4) Somewhat oppose | |
